# Long-term outcome and toxicity of hypofractionated stereotactic body radiotherapy as a boost treatment for head and neck cancer: the importance of boost volume assessment

**DOI:** 10.1186/1748-717X-7-85

**Published:** 2012-06-12

**Authors:** Dong Soo Lee, Yeon Sil Kim, Jae Seok Cheon, Jin Ho Song, Seok Hyun Son, Ji Sun Jang, Young Nam Kang, Jing Hyoung Kang, So Lyoung Jung, Ie Ryung Yoo, Hong Seok Jang

**Affiliations:** 1 Department of Radiation Oncology, Yonsei Cancer Center, Severance Hospital, Yonsei University Heath System, Seoul, Republic of Korea; 2 Department of Radiation Oncology, College of Medicine, The Catholic University of Korea, Seoul, Republic of Korea; 3 Department of Medical Oncology, College of Medicine, The Catholic University of Korea, Seoul, Republic of Korea; 4 Department of Radiology, College of Medicine, The Catholic University of Korea, Seoul, Republic of Korea; 5 Department of Nuclear Medicine, College of Medicine, The Catholic University of Korea, Seoul, Republic of Korea; 6 Department of Radiation Oncology, Seoul St. Mary’s Hospital, College of Medicine, The Catholic University of Korea, 505 Banpo-dong, Seocho-gu, Seoul, 137-701, Republic of Korea

**Keywords:** Boost, Head and neck cancer, Hypofractionation, Stereotactic body radiotherapy

## Abstract

**Background:**

The aim of this study was to report the long-term clinical outcomes of patients who received stereotactic body radiotherapy (SBRT) as a boost treatment for head and neck cancer.

**Materials and methods:**

Between March 2004 and July 2007, 26 patients with locally advanced, medically inoperable head and neck cancer or gross residual tumors in close proximity to critical structures following head and neck surgery were treated with SBRT as a boost treatment. All patients were initially treated with standard external beam radiotherapy (EBRT). SBRT boost was prescribed to the median 80% isodose line with a median dose of 21 (range 10–25) Gy in 2–5 (median, 5) fractions.

**Results:**

The median follow-up after SBRT was 56 (range 27.6 − 80.2) months. The distribution of treatment sites in 26 patients was as follows: the nasopharynx, including the base of the skull in 10 (38.5%); nasal cavity or paranasal sinus in 8 (30.8%); periorbit in 4 (15.4%); tongue in 3 (11.5%); and oropharyngeal wall in 1 (3.8%). The median EBRT dose before SBRT was 50.4 Gy (range 39.6 − 70.2). The major response rate was 100% with 21 (80.8%) complete responses (CR). Severe (grade ≥ 3) late toxicities developed in 9 (34.6%) patients, and SBRT boost volume was a significant parameter predicting severe late complication.

**Conclusions:**

The present study demonstrates that a modern SBRT boost is a highly efficient tool for local tumor control. However, we observed a high frequency of serious late complications. More optimized dose fractionation schedule and patient selection are required to achieve excellent local control without significant late morbidities in head and neck boost treatment.

## Introduction

Radiation therapy is an established loco-regional treatment for locally advanced head and neck cancers [1,2]. Although excellent results have been achieved with advances in management schemes [2-4], local recurrence continues to be a therapeutic challenge and one of the major causes of treatment failure, especially in locally advanced squamous cell carcinomas of the head and neck [5,6]. Moreover, in cases of nasal cavity, paranasal sinus (PNS) or nasopharyngeal carcinoma (NPC), which are neighboring critical structures (e.g., brainstem and optic apparatus), dose escalation with conventional external beam radiotherapy (EBRT) is limited by the radiation tolerance of the adjacent normal tissues.

It has been generally accepted that a dose–response relationship exists between the applied radiation dose and local tumor control [7,8]. Furthermore, absolute local control is one of the essential prognostic indicators for maintaining excellent long term clinical results.

The CyberKnife (Accuray, Sunnyvale, CA) was originally developed for high-precision stereotactic radiotherapy. The feasibility, safety, and success in terms of excellent outcomes of the CyberKnife (CK) treatment as a boost, re-irradiation or palliative modality have been previously reported [9-12]. However, long-term safety profiles and normal tissue dose-volume constraints for modern hypofractionated stereotactic body radiotherapy (SBRT) are still not documented.

Our interim preliminary data have shown that SBRT boosting might improve local tumor control through increased radiation dosing with acceptable acute toxicity. The purpose of the current study is to report the long-term clinical outcome and toxicity of using SBRT as a boost treatment for locally advanced head and neck cancer.

## Materials and methods

### Study design and patient population

Patients who were treated with radiosurgery boosting using the CK between March 2004 and July 2007 with one the following characteristics were enrolled as subjects into this study (*N* = 26): patients with locally advanced or medically inoperable head and neck cancers or patients with residual tumors that were adjacent to the critical structures after surgery in the head and neck region. More detailed characteristics of included patients were as follows: locally advanced NPC patients who needed high dose of radiation for local tumor control, PNS or nasal cavity cancer patients whose tumors located in close proximity to the orbital structure, patients with minor salivary gland cancer arising from orbital structures in which high radiation delivery is not practically possible with conventional three-dimensional (3-D) conformal approach, and medically inoperable T2-T3 category of oral tongue cancer patients who needed an efficient dose escalation tool after EBRT of 45 Gy instead of brachytherapy boost.

We obtained Institutional Review Board approval for this retrospective study. All of the patients were initially treated with standard therapies, including 3-D conformal EBRT. SBRT boost was administered to the gross tumor volume (GTV) area 2 weeks after the completion of the initial EBRT treatment. The SBRT schedule typically consisted of dose between 10 and 25 Gy in 3 to 5 fractions, and we adjusted the SBRT dose and fractionation schedule according to the initial tumor site and type, modality of initial treatment, and previous EBRT dose.

### CyberKnife treatment procedures

The CK treatment procedure has been fully described previously [9,10]. In almost all cases, we performed image fusion of pre-treatment 1-mm slice magnetic resonance imaging (MRI) images with computed tomography (CT) images that had been taken in the same positions to define target volumes and critical structures. Although we could achieve tumor shrinkage after EBRT in NPC patients, involved skull base and parapharyngeal mucosa often had residual lesions. Therefore, we included aforementioned areas as a SBRT boost volume. In non-NPC patients, residual tumors neighboring critical organs after EBRT (with or without previous debulking surgery) were defined as a SBRT boost volume. The planning target volume (PTV) was defined as a radiographic GTV with a 1- to 2-mm margin 3-D expansion. The PTV was slightly modified by the surrounding critical structures, such as the optic chiasm, optic nerve, brainstem, lens, globe, and spinal cord. Dose constraints to normal structures were determined based on the EBRT dose and tumor locations. These constraints included the total doses (EBRT and SBRT dose) to the globe of <45 − 50 Gy, optic nerves/chiasm of <54 − 60 Gy, and brainstem of <54 − 60 Gy, unless there were direct tumor invasion into these structures.

During the time period of this treatment, there was no firmly established dose constraints guideline for normal organs in hypofractionated RT. We planned to deliver smallest possible doses to these organs; and higher doses to the limited volumes of these structures were selectively allowed to appropriately cover the target volumes. A total of 3 to 5 fractions were delivered for 3 to 5 consecutive days without inter-fractional break times. SBRT dose-fractionation schedules used in our study are summarized in Table 1.

**Table 1:** SBRT dose-fractionation schedules

**Number of patients**	**Fractions**	**Dose (Gy)**	**BED_10_ (Gy)**	**BED_3_ (Gy)**
8	5	25	37.5	66.7
6	3	21	35.7	70
5	5	20	28	46.7
1	3	24	43.2	88
1	4	24	38.4	72
1	5	22.5	32.6	56.3
1	3	18	28.8	54
1	4	18	26.1	45
1	2	16	28.8	58.7
1	3	10	13.2	20.8

BED, Biologically equivalent dose.

### Follow-up and response assessment

The patients were followed up at one-, three-, and six-month intervals after SBRT treatment during the first year; at every three- to six-month intervals during the second year; and at every six-month interval thereafter. During the follow-up periods, physical examination and nasopharyngoscopy, in addition to CT, MRI or 18 F-fluorodeoxyglucose (FDG) positron emission tomography CT (PET-CT) imaging studies, were routinely performed at usually every three-month after the completion of treatment for the first two years and then six-month interval thereafter. Biopsies were performed when clinically indicated.

The disappearance of tumors as evidenced by contrast-enhanced CT, MRI, nasopharyngoscopy, and fibrotic changes that were inconsistent with the presence of tumors with no evidence of progression in serial imaging studies were regarded as a complete response (CR). A partial response (PR) was determined in the same manner as a CR, and the tumor had to be reduced by >50% but not completely gone.

Acute and late toxicities were assessed using the National Cancer Institute Common Toxicity Criteria (NCI CTCAE), version 4.0, and the Radiation Therapy Oncology Group (RTOG) late toxicity criteria, respectively.

### Statistical analysis

All statistical analyses were performed using SPSS software (version 12.0, SPSS Inc., Chicago, IL, USA). Fisher’s exact test and the Mann–Whitney U test were used to compare variable distributions. Loco-regional recurrence-free rate (LRRFR) and overall survival (OS) were estimated using Kaplan-Meier curves, and survival differences were assessed using log-rank tests. The Cox proportional hazards regression model was used to identify independent prognostic factors. All statistical analyses were calculated based on the two-sided test, and a p-value < 0.05 was considered to be significant.

## Results

### Patient characteristics

The median follow-up after SBRT treatment was 56 (range 27.6 − 80.2) months, and 13 (50%) patients had died by the time of the analysis. A complete history, physical examination, CT, MRI, PET-CT and nasopharyngoscopy were routinely performed before the initial treatments, and none of the patients showed evidence of distant metastasis (DM) at the time of the initial diagnoses. All patients had an Eastern Cooperative Oncology Group (ECOG) scale score <2 with acceptable bone marrow, liver and renal functions. Stage was based on clinical staging system in NPC patients and patients who did not undergo surgery, and pathologic staging system in patients who underwent initial debulking surgery. Patient, tumor, and treatment characteristics are described in Table 2.

**Table 2:** **Patient, tumor, and treatment characteristics (*N*=26)**

**Characteristics**	**Disease group [No. (%)]**	
	**NPC (*n*=10)**	**Others (*n*=16)**
Age (year)		
Median	54	58.5
Range	31-67	10-83
Gender		
Male:Female	8 (80): 2 (20)	12 (75): 4 (25)
Sites treated with SBRT		
Nasopharynx and base of skull	10 (100)	
Nasal cavity and PNS		8 (50)
Periorbit		4 (25)
Tongue		3 (18.8)
Oropharyngeal wall		1 (6.2)
Pathology		
Type I NPC	2 (20)	
Type II NPC	8 (80)	
Squamous cell carcinoma		7 (43.8)
Adenoid cystic carcinoma		5 (31.3)
Chondrosarcoma		2 (12.5)
Malignant melanoma		1 (6.2)
Poorly differentiated adenocarcinoma		1 (6.2)
Tumor T stage		
T4	7 (70)	7 (43.8)
T3	2 (20)	4 (25)
T2	1 (10)	5 (31.2)
Tumor N stage		
N2	3 (30)	1 (6.3)
N1	6 (60)	2 (12.5)
N0	1 (10)	13 (81.2)
Previous debulking surgery		
Yes:No	0 (0):10 (100)	11(68.8): 5 (31.2)
Combined chemotherapy		
Yes:No	10 (100): 0 (0)	9 (56.3): 7 (43.7)
SBRT GTV (cc)		
Median	45.3	19.4
Range	21.3-69.4	6.9-66.8
Prescription isodose line (%)		
Median	80	80
Range	70-80	70-85
EBRT dose (Gy)		
Median	59.4	47.5
Range	50.4-70.2	39.6-59.4
SBRT dose (Gy)		
Median	21	22.5
Range	18-25	10-25
SBRT fractional dose (Gy)		
Median	6.5	5
Range	5-7	3-8
Cumulative dose in BED_10_ (Gy)		
Median	103.7	90.6
Range	92-118.5	72.7-107.6

SBRT, stereotactic body radiotherapy; PNS, paranasal sinus; NPC, nasopharyngeal cancer; T, tumor; N, node; GTV, gross tumor volume; EBRT, external beam radiotherapy; BED_10_, biologically equivalent dose in α/β ratio of 10.

### Tumor and treatment characteristics

The median EBRT dose before SBRT was 50.4 (range 39.6 − 70.2) Gy. The median SBRT dose was 21 (range 10–25) Gy in 2–5 (median 5) fractions. The median PTV coverage was 98.2% (range 93.8 − 100%), and the median target volume was 28.2 (range 6.9 − 69.4) cc. The previous EBRT and SBRT doses were converted to biologically equivalent doses (BEDs) based on a linear-quadratic (LQ) equation to sum the total radiation dose because the dose per fraction differed among patients. The alpha/beta (α/β) ratio was assumed to be 10 for the acute responding tissue and tumors and 3 for late-responding tissues. The cumulative BED_10_ ranged from 72.7 to 118.5 (median 94.9) Gy. When converted to a normalized total dose (NTD) of 1.8 Gy, the SBRT NTD_1.8Gy, α/β=10_ ranged from 22.1 to 36.6 (median 30.3) Gy, and the cumulative NTD_1.8Gy_ ranged from 61.7 to 100.5 (median 79.9) Gy.

Combined chemotherapy was used in 19 (73.1%) patients. Among these patients, 15 (57.7%) had received concurrent chemotherapy, whereas the remaining 4 (15.4%) had received sequential chemotherapy. Debulking surgery was performed in 11 (42.3%) non-NPC patients prior to EBRT.

Treatment compliance was excellent, and all patients completed their planned treatments; however, in one NPC patient, treatment was delayed for approximately 1 week because of severe nausea during the SBRT procedure.

Based on combinatorial imaging studies or biopsy, 21 (80.8%) patients achieved CR, whereas 5 (19.2%) patients achieved PR. As a result, the major response (CR + PR) rate was 100%. The median time to the maximal response was 2 (range 0.3 − 7.4) months.

### Patterns of failure and survival assessment

During the follow-up periods, a total of 8 (30.7%) recurrences initially developed at the following sites: 2 local failures, 1 regional failure and 5 DM. Two local failures were infield recurrences, and 1 regional failure was an internal jugular chain recurrence that was adjacent to the primary target volume. The median time to the LRR and DM was 5.5 (range 4.6 − 13.3) and 9.3 (range 1.0 − 13.3) months, respectively. Two infield recurrences from the two NPC patients and 1 regional recurrence from a tonsillar carcinoma patient developed. Two bone metastases occurred from NPC, 1 bone metastasis from lacrimal gland ACC, 1 skin metastasis from malignant melanoma, and 1 liver metastasis from maxillary sinus adenocarcinoma.

The OS duration was assessed from the SBRT completion date to the date of death or the patient’s last visit. For the entire study population, the median OS duration was 31.5 (range 4.5 − 73.6) months. The 2- and 5-year actuarial OS rates for the entire population were 61.5% and 46.2%, respectively. The 1- and 2-year actuarial LRRFR for the entire population were 91.4% and 86.3%, respectively.

### Toxicity

A total of 13 patients experienced acute toxicities. Mild to moderate (grade ≤ 2) toxicities developed in six patients who presented with mucositis, emesis, or conjunctivitis. Severe (grade ≥ 3) mucositis developed in seven patients, including six grade 3 and one who was grade 4.

The frequency of late toxicities was unacceptably high. Grade 1–2 manageable toxicities developed in nine patients, including 1 minimal but prolonged mucosal ulcer over 1 year, 3 cataracts, 3 nasolacrimal duct stenoses or occlusions, 1 soft tissue and skin fibrosis, and 1 unhealed wound dehiscence.

Severe (grade ≥ 3) late toxicities developed in nine (34.6%) patients. The detailed characteristics of these nine patients who experienced late toxicities are listed in Table 3. Four cases developed within the target volumes, 5 cases developed in critical structures, and 1 was a combined case. The median elapsed time of late toxicity after SBRT was 20.9 (range 4.4 − 32.4) months. More than 2 mixed late complications developed in three patients. In nine patients, eight had achieved CR and one PR in their treatment courses, and seven patients had been treated with concurrent chemoradiation prior to their SBRT procedures. Bone or soft tissue necrosis developed within a relatively a short period of time (4.4 − 8.5 months) in comparison to the development of other neuronal complications. We investigated whether patient and treatment characteristics would be associated with late complications. Among the variables, a large GTV in SBRT was only significantly associated with late toxicities (p = 0.038), and SBRT fractional dose was also a marginally significant factor (p = 0.058) (Table 4).

**Table 3:** Nine cases of severe late complications

**Patient (age/sex)**	**Treatment site**	**Elapsed time (months)**	**SBRT dose schedule (total dose/fraction)**	**Response**	**Cumulative BED_10_ (Gy)**	**Late toxicity (RTOG late toxicity grade)**
31/M	NPC, Lt. skull base	24.4	21/3	CR	101.5	Pontine necrosis (G5^*^)
48/M	NPC, Lt. skull base	8.5	21/3	PR	105.8	Lt. base of skull bone and soft tissue necrosis (G4)
48/M	NPC, Rt. skull base	25.9	25/5	CR	107.6	Pontine necrosis (G3)
48/M	NPC, Lt. skull base	4.4, 6.8	21/3	CR	118.5	Lt. nasopharyngeal wall soft tissue necrosis (G4), temporal lobe necrosis (G3)
55/M	SCC, Rt. BOT	15.8	16/2	CR	94.6	Mucosal ulcer and necrosis (G4)
82/M	SCC, Lt. BOT	20.9	24/3	CR	96.3	Unhealing mucosal ulcer and bleeding (G4)
36/F	ACC, Rt. lacrimal gland	30.0, 32.4, 30	25/5	CR	90.6	Radiation retinopathy (G3), temporal lobe necrosis (G3), NVG (G3)
41/F	ACC, Lt. lacrimal gland	28.5, 28.5	20/5	CR	87.5	Radiation retinopathy (G3), NVG (G3)
59/M	ACC, Rt. orbit	15.1	25/5	CR	90.6	Optic neuropathy (G3)

^*^Pontine necrosis was the definite cause of death

SBRT, stereotactic body radiotherapy; BED_10_, biologically equivalent dose in α/β ratio of 10; M, male; NPC, nasopharyngeal carcinoma; CR, complete response; PR, partial response; SCC, squamous cell carcinoma; BOT, base of tongue; F, female; ACC, adenoid cystic carcinoma; NVG, neovascular glaucoma.

**Table 4:** Risk factor analysis of developing grade ≥3 late toxicities

**Characteristics**	**Grade≥3 late toxicities**		
	**No (*n*=17)**	**Yes (*n*=9)**	***p***
Age			0.218^*^
<55	6 (35.3)	6 (66.7)	
≥55	11 (64.7)	3 (33.3)	
Gender			1.000^*^
Male	13 (76.5)	7 (77.8)	
Female	4 (23.5)	2 (22.2)	
Surgery			0.683^*^
No	9 (52.9)	6 (66.7)	
Yes	8 (47.1)	3 (33.3)	
Response			1.000^*^
CR	13 (76.5)	8 (88.9)	
PR	4 (23.5)	1 (11.1)	
Histology			0.692^*^
NPC	6 (35.3)	4 (44.4)	
Non-NPC	11 (64.7)	5 (55.6)	
EBRT dose (Gy)			0.525^†^
Median (range)	50.4 (39.6-60)	55.8 (45-70.2)	
SBRT BED_10_ (Gy)			0.312^†^
Median (range)	35.7 (13.2-38.4)	35.7 (28-43.2)	
Total BED_10_ (Gy)			0.287^†^
Median (range)	93.8 (72.7-107.6)	96.3 (87.5-118.5)	
SBRT GTV (cc)			0.038^†^
Median (range)	21 (6.9-69.4)	47.7 (20.9-66.8)	
SBRT fraction number			0.339^†^
Median (range)	5 (3-5)	3 (2-5)	
SBRT fractional dose (Gy)			0.058^†^
Median (range)	5 (3-7)	7 (4-8)	

^*^Fisher’s exact test; ^†^Mann-Whitney U test

CR, complete response; PR, partial response; NPC, nasopharyngeal carcinoma; EBRT, external beam radiotherapy; SBRT, stereotactic body radiotherapy; BED_10_, biologically equivalent dose in α/β ratio of 10; GTV, gross tumor volume.

**Figure 1:**
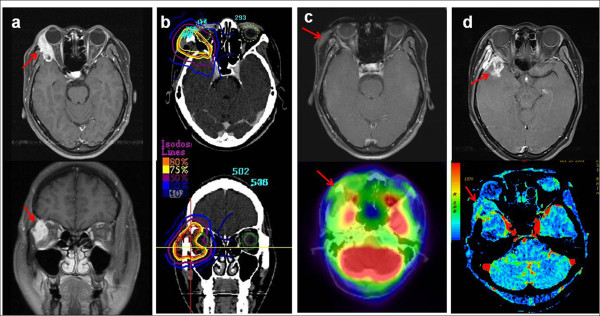
**Radiation retinopathy, neovascular glaucoma and temporal lobe necrosis after SBRT.** This patient was a 36-year-old female with adenoid cystic carcinoma (ACC) of the right lacrimal gland with intraorbital invasion (a). She underwent right orbitotomy and neck dissection followed by EBRT at 45 Gy and SBRT at 25 Gy in 5 fractions (b) and achieved CR in the response evaluation (c). Two years after SBRT, a cataract operation (Phaco + PCL) of the right eye was performed. She experienced progressive vision loss in the right eye and was diagnosed with radiation retinopathy and neovascular glaucoma in an ophthalmologic exam 30 months after SBRT. Follow-up serial perfusion CT and functional MRI of the brain indicated right temporal lobe necrosis of the brain (d).

**Figure 2:**
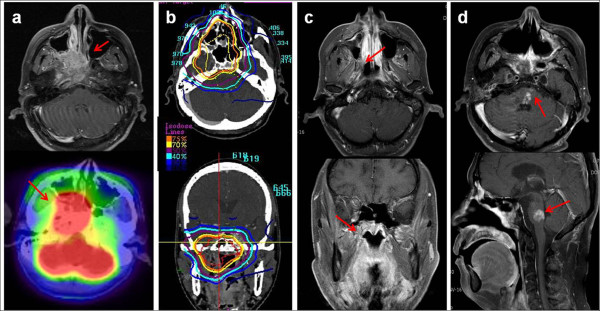
**Pontine necrosis after SBRT.** This patient was a 48-year-old male with type I NPC (a). He was treated with cisplatin-based concurrent chemoradiation via EBRT up to 59.4 Gy followed by CK-RS at 25 Gy in 5 fractions (b) and achieved CR in the response evaluation (c). Pontine necrosis developed 25.9 months after SBRT (d). He experienced various neurologic symptoms, including dizziness, gait disturbance, hoarseness, limb weakness, dysarthria and other cranial nerve signs and was still under rehabilitation and supportive care for debilitating symptoms at the time of analysis.

**Figure 3:**
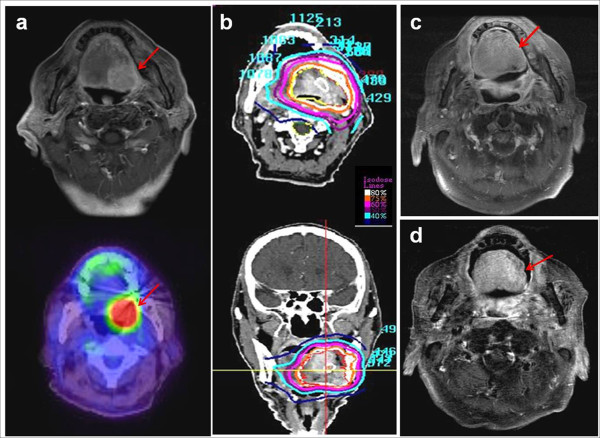
**Non-healing mucosal ulcer and bleeding after SBRT.** This patient was an 82-year-old male with cancer of the base of the tongue. He was diagnosed with left posterolateral SCC of the base of the tongue (T2N0M0) (a) and was treated with EBRT at 45 Gy followed by CK-RS boost at 24 Gy in 3 fractions (b). He achieved CR in the response evaluation (c); however, he suffered from prolonged chronic mucositis and poor oral intake and finally died from asphyxia that was caused by a massive ulcer bleeding 22.6 months after SBRT, despite medical treatment (d).

**Figure 4:**
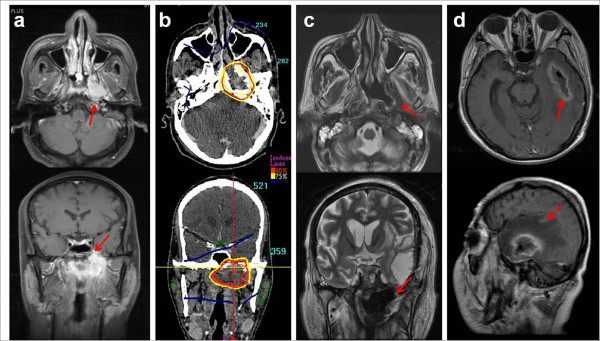
**Left nasopharyngeal wall soft tissue necrosis and temporal lobe necrosis after SBRT.** This patient was a 48-year-old male with type I NPC (T4N2M0) (a). He was treated with cisplatin-based concurrent chemoradiation via EBRT up to 70.2 Gy followed by SBRT at 21 Gy in 3 fractions due to persistent disease in the left skull base (b). Although he achieved CR and no loco-regional or distant metastasis occurred, left nasopharyngeal wall soft tissue necrosis (c) and temporal lobe necrosis (d) developed 4.4 and 7.2 months after SBRT, respectively. Temporal lobe necrosis and abscess formation, which might have arisen from adjacent nasopharyngeal soft tissue necrosis, were confirmed by surgical specimen pathology. He finally died 9.3 months after the SBRT treatment.

There were 2 deaths due to SBRT-related late toxicities. One patient suffered from prolonged poor oral intake due to a non-healing mucosal ulcer, and he finally died from a poor general condition and asphyxia caused by massive oral bleeding. Another patient died from neurologic deteriorations arising from pontine necrosis from which he experienced a number of neurological symptoms, including hoarseness, shoulder pain, limb weakness and aspiration, and finally expired 38.2 months after the SBRT procedure. Figures 1, 2, 3 and 4 show representative cases of late complications.

### Dosimetric analysis of late complications

The results of detailed dose-volume analyses of 4 major late complications are described in Table 5. The median EBRT and SBRT prescribed doses were 50.4 (range 45–70.2) and 25 (range 20–25) Gy, respectively. We estimated the D_30%_ (a dose covering 30% of the organ volume) and D_50%_ in the EBRT course and D_10%_, D_30%_, D_50%_, V_50%_ (an organ volume that received 50% of the total dose) and V_70%_ in the SBRT course to evaluate partial volume dosimetry. The Dmax (maximum dose) for the organ at risk (OAR) was higher than the prescribed dose in 6 out of 7 cases. The SBRT V_50%_ was higher than 20 − 30% in all of the pontine necrosis, optic retinopathy and optic neuropathy cases.

**Table 5:** Dose-volume analysis of 4 major late complications

**Complications**	**OAR volume**	**EBRT dose**	**SBRT dose**	**EBRT D_30%_**	**EBRT D_50%_**	**SBRT Dmax at OAR**	**SBRT D_10%_**	**SBRT D_30%_**	**SBRT D_50%_**	**SBRT V_50%_**	**SBRT V_70%_**
	(cc)	(Gy)	(Gy/Fx)	(Gy)	(Gy)	(Gy)	(Gy)	(Gy)	(Gy)	(%)	(%)
Pontine necrosis (Brainstem)											
1	24.67	59.4	25/5	43.5	40.9	24.6	19	15.4	12.8	23.5	0.7
2	23.55	55.8	21/3	36.2	19.7	27.3	24.2	20.1	16.0	54.3	24.9
Temporal lobe necrosis (Temporal lobe)											
1	63	45	25/5	48.1	45.3	28.4	17.9	10.3	6.2	11.2	4.4
2	66.02	70.2	21/3	37.4	11.7	25.0	16.5	8.9	4.9	18.8	5.7
Optic retinopathy (Eyeball)											
1	10.7	45	25/5	45.1	45.1	27.7	23.7	18.4	13.3	35.9	12.8
2	11.2	50.4	20/5	52.1	49	22.5	21.1	18	13.5	50.6	25.9
Optic neuropathy (Optic nerve)											
1	0.483	45	25/5	41.3	40.7	27.8	27.3	27.0	26.7	100	97.2

OAR, organ at risk; EBRT, external beam radiotherapy; SBRT, stereotactic body radiotherapy; Fx, fraction; D_x%_, dose covering x% of total volume; Dmax, maximum dose; V_x%,_ volume receiving x% of prescribed dose.

## Discussion

The present study showed that modern SBRT boost technique was highly efficacious dose escalation modality in terms of local control. However, we also observed high late complications.

In the current study, the major response rate with early local control was 100%, including 21 (80.8%) CR. Despite the treated tumors having heterogeneous compositions and treatment characteristics, the frequency of infield local recurrence was low (7.7%) after long-term follow-up. The importance of improving local control is demonstrated in the present study as well as in previous reports, which indicates that local recurrence exacerbates and deteriorates the OS [5]. Due to the fact that the DM (19.2%) was the major pattern of failure, the results support the need for more effective systemic chemotherapy or targeted therapy in patients who are treated with highly ablative tools for local treatment.

In terms of toxicity profiles, our current study demonstrates somewhat unfavorable results. Acute complications (27%) as well as severe late complications (34.6%) were observed to unexpectedly develop at high frequencies. Although the Quantitative Analysis of Normal Tissue Effects (QUANTEC) [13], which is a new dose-volume parameter in late complications, have been recently reported, they have demonstrated a disagreement among existing models and lack significant published data, particularly in the context of hypofractionated RT. Accordingly, there is a lack of firmly established dose-volume criteria regarding late toxicities for hypofractionated SBRT at present. In our study, seven (77.8%) out of nine patients who developed severe late complications had received prior concurrent chemoradiation, and SBRT was continuously employed after only 2 weeks. Therefore, aggressive treatment within a short period might lead to these unacceptable toxicities. There were 2 types of late toxicities in our data: toxicity within target volumes and toxicity in neighboring normal structures. Because one case of early death were caused by sustained chronic bleeding-related aspiration, prolonged mucositis or unhealed ulcers, late toxicities arising from target volumes or swallowing structures should not be overlooked. Consequential late effects might be another related factor, as some patients experienced severe early toxicity. Late complications in critical organs developed within relatively consistent time intervals after SBRT. Pontine necrosis, radiation retinopathy and NVG progressed in around 2–3 years after SBRT.

Debus et al*.*[14] reported that the mean time to onset of brainstem symptoms was 17 (range, 4.5 − 92) months in patients who developed brainstem toxicity after photon and proton radiotherapy for chondrosarcoma and skull base tumors. Symptoms appeared within 3 years in 89.5% of patients, and there was a trend of a higher grade of toxicity in patients who had late symptom onsets. In most earlier series by Jiang et al. [15], the actuarial incidence of symptomatic retinopathy and neuropathy constantly increased even after 5 years in patients who had been treated with doses higher than 60 Gy. The incidence of TLN was particularly high in a series by Hara et al. [10] in which the authors prospectively performed SBRT boosting in a single fraction after EBRT for patients with locally advanced NPC. Although the overall local control was excellent (98% at 5 years), 12% of patients developed TLN from 18 to 97 months after completion of SBRT boost. As shown in previous data, toxicity results in the scope of hypofractionation RT were scarce and insufficient to propose a standardized guideline.

Given the high α/β ratio in the majority of head and neck tumors and the low α/β ratio in the surrounding normal organs, hypofractionation with extremely high fraction sizes might not be a relevant dose scheme. Moreover, a dose calculation method using a simple dose summation of BED based on the LQ-model is a controversial matter [16,17] and has some limitations because, hypothetically, the normal tissue response to a high dose per fraction is potentiated by direct cytotoxic damage and vascular/stromal damage [16].

From our data, we can conclude that the existence of high-grade early toxicity, aggressive treatment without sufficient break time, a large treatment volume and high fractional dose are consistently associated with late complications. The majority of early deaths without evidence of recurrent disease were observed to be related to persistent swallowing problems, chronic mucositis, or the impairment of neuronal functions (e.g., gag reflex). In addition, there were several cases of late complications that derived from eye and neuronal structures. Although rapid dose fall-off was seen in the high-dose area (Table 5), there were several cases with high V_50%_ in our study. Therefore, we need to more strictly reduce high-dose volumes in these organs to avoid severe late complications.

The main advantage of the current study is the achievability of long-term follow-up in the entire patient cohort. The results from the current series were concordant with the early experience of hypofractionated RT in the historical data with regard to the possibility of late unfavorable sequelae [18]. We have changed our treatment planning to decrease the fractional dose and are currently applying the SBRT boost technique only in selective cases with limited volumes in the head and neck area because a large treatment volume and a high fractional dose were the significant related parameters that were predictive of the risk of developing late toxicities in our study.

In conclusion, the present study shows that the modern SBRT boost technique is a highly effective treatment tool for local tumor control in extracranial head and neck cancers. However, we observed serious late complications related with high boost volume and large fractional dose. Although generally considered to be a safe modality, hypofractionated SBRT boost should be employed with caution in selected patients due to its potentially hazardous effects in head and neck regions. Further validations for determining the appropriate biological model in the field of hypofractionation RT are warranted from future clinical studies.

## Abbreviations

PNS, Paranasal sinus; NPC, Nasopharyngeal cancer; EBRT, External beam radiotherapy; CK, Cyberknife; SBRT, Stereotactic body radiotherapy; GTV, Gross tumor volume; MRI, Magnetic resonance imaging; CT, Computed tomography; PTV, Planning target volume; FDG, Fluorodeoxyglucose; PET, Positron emission tomography; CR, Complete response; PR, Partial response; LRRFR, Loco-regional recurrence-free rate; OS, Overall survival; DM, Distant metastasis; ECOG, Eastern Cooperative Oncology Group; BED, Biologically equivalent doses; LQ, Linear-quadratic; NTD, Normalized total dose; OAR, Organ at risk.

## Competing interests

The authors declare that they have no competing interest.

## Authors’ contributions

DSL carried out the data collection, participated in the study design and manuscript writing; YSK treated patients, participated in the study design and coordination, and helped collect data; JSC, JHS, SHS, JSJ, YNK and HSJ helped the data collection and analysis; DSL, YSK and JSC carried out the data and statistical analysis; JHK treated patients as a medical oncologist; SLJ and IRY interpreted imaging studies as a radiologist and nuclear medicine doctor. All authors read and approved the final manuscript.
